# Explore the changes of intestinal flora in patients with coronavirus disease 2019 based on bioinformatics

**DOI:** 10.3389/fcimb.2023.1265028

**Published:** 2023-10-13

**Authors:** Gangding Huang, Yanning Mao, Weiwei Zhang, Qi Luo, Rong Xie, Dongmei Huang, Yumei Liang

**Affiliations:** Department of Gastroenterology, the Fifth Affiliated Hospital of Guangxi Medical University, Nanning, China

**Keywords:** COVID-19, 16S rRNA gene sequencing, intestinal flora, bioinformatics analysis, amplicon sequence variant

## Abstract

**Background:**

Studies have revealed that there were significant changes in intestinal flora composition in patients with coronavirus disease 2019 (COVID-19) compared to non-COVID-19 patients, regardless of whether they were treated with medication. Therefore, a comprehensive study of the intestinal flora of COVID-19 patients is needed to further understand the mechanisms of COVID-19 development.

**Methods:**

In total, 20 healthy samples and 20 COVID-19 samples were collected in this study. Firstly, alpha diversity and beta diversity were analyzed to assess whether there were difference in species richness and diversity as well as species composition between COVID-19 and control groups. The observed features index, Evenness index, PD index, and Shannon index were utilized to measure alpha diversity. The principal coordinates analysis (PCoA) and non-metric multidimensional scaling (NMDS) were performed to analyzed beta diversity. Linear discriminant analysis Effect Size (LEfSe) was utilized to analyze the variability in the abundance of bacterial taxa from different classification levels. The random forest (RF), Least absolute shrinkage and selection operator (LASSO), and univariate logistic regression were utilized to identify key Amplicon Sequence Variant (ASVs). Finally, the relevant networks of bacterial taxa were created in COVID-19 and control groups, separately.

**Results:**

There were more species in the control group than in COVID-19 group. The observed features index, Shannon index, and Evenness index in the control groups were markedly higher than in the COVID-19 group. Therefore, there were marked variations in bacterial taxa composition between the COVID-19 and control groups. The nine bacterial taxa were significantly more abundant in the COVID-19 group, such as g-Streptococcus, *f-Streptococcaceae, o-Lactobacillales, c-Bacilli* and so on. In the control group, 26 bacterial taxa were significantly more abundant, such as *c-Clostrjdia, o-Oscillospirales, f-Ruminococcaceae*, etc. The 5 key ASVs were obtained through taking the intersection of the characteristic ASVs obtained by the three algorithms, namely ASV6, ASV53, ASV92, ASV96, and ASV105, which had diagnostic value for COVID-19. The relevance network in the control group was more complex compared to the COVID-19 group.

**Conclusion:**

Our findings provide five key ASVs for diagnosis of COVID-19, providing a scientific reference for further studies of COVID-19.

## 1 Introduction

Coronavirus disease 2019 (COVID-19) is a severe acute respiratory syndrome caused by coronavirus 2 (SARS-CoV-2). SARS-CoV-2 primarily infects the lungs, and can cause fever with cough and dyspnea, which are the most common manifestations in patients (Guan et al., 2020). Although most cases of COVID-19 are mild, severe cases can lead to respiratory failure or death (Onder et al., 2020). Further, COVID-19 is extremely harmful, spreads quickly and is highly pathogenic (Yang et al., 2022). According to the World Health Organization (WHO), as of February 1, 2022, there have been 379,223,560 COVID-19 infections and 5,693,245 deaths worldwide (Alharbi et al., 2022). It is found that COVID-19 can lead to dysbacteriosis of respiratory flora. For example, tract microbiota dysbiosis of the upper respiratory tract in critically ill patients with COVID-19 is more severe than in patients without COVID-19, and the oropharyngeal microbiome in COVID-19 patients is significantly disrupted and is associated with disease severity. And meanwhile, intubated patients with COVID-19 have low diversity in endotracheal samples and show frequent growth of potential respiratory pathogens, particularly staphylococcic (Merenstein et al., 2022).

It is worth that the initial symptoms of COVID-19 patients are mainly fever, fatigue, and dry cough, but clinical studies have also found that SARS-CoV-2 can also cause gastrointestinal symptoms such as diarrhea, abdominal pain, nausea, and vomiting (Du et al., 2020). Further, diarrhea is detected as a common extrapulmonary symptom in people with COVID-19, and infectious SARS-CoV-2 has been detected in stool samples as well (Gu et al., 2020). Among about 50 percent of patients, SARS-CoV-2 RNA can still be detected in stool samples when the virus is no longer detected in the respiratory tract (Xiao et al., 2020). This suggests that the gastrointestinal tract may be the site of active viral replication, and that viruses may directly interfere with local ecosystems in the gut, leading to disturbances in the gut flora.

Intestinal flora is an important factor in regulating intestinal homeostasis (Peters et al., 2018; Park et al., 2019). Microbial imbalances in the body can lead to a variety of diseases and immune responses (Johnson et al., 2016; Golonka et al., 2019). The COVID-19 pandemic-related studies have shown that, compared with non-COVID-19 patients, the composition of the gut microbiota of COVID-19 patients changed significantly, regardless of whether the patients received medication (Gaibani et al., 2021; Yeoh et al., 2021), indicating a potential relationship between gut microbiota and SARS-CoV-2 infection (Cao et al., 2021). Besides, enrichment of certain bacteria, such as Coenobacterium and Clostridium, is considered positively correlated with the severity of COVID-19 through shotgun sequencing (Yeoh et al., 2021). Considering previous studies have found an association between certain respiratory diseases and diseases of the digestive tract (Keely et al., 2012), while, the diagnostic potential of the microbiota profiles for predicting COVID-19 and healthy controls has not been systematically explored yet. Therefore, the study of intestinal flora in patients with COVID-19 diagnosis may be a new breakthrough point.

Given that the concerns regarding the 20% false negative rate associated with RT-PCR-based nucleic acid detection and the promising possibilities of bioinformatics analysis in the identification of biomarkers as non-invasive diagnostic tools for diagnosing COVID-19 (Ren et al., 2021; Aishwarya and Gunasekaran, 2022), on the basis of twenty patients with confirmed COVID-19 and 20 non-COVID-19 patients, we assessed the differences in gut microbiome as well as functional characteristics of bacterial taxa between the two by 16S rRNA sequencing, and further screened the key diagnostic ASVs using three machine algorithms, in order to provide a new theoretical basis for the diagnosis and prevention of COVID-19. The microbiota diversity of the control group was higher than that of the COVID-19 group. Results of KEGG pathway predictions differed between COVID-19 and control groups as well. Further, a model was constructed by five critical ASVs with COVID-19 diagnostic values. Moreover, the correlation between bacterial taxa in the control group was more complex compared to that in the COVID-19 group. This study comprehensively studies the relationship between intestinal flora and COVID-19, and provides new diagnostic ideas for further understanding of the mechanism of COVID-19 occurrence and development.

## 2 Materials and methods

### 2.1 Subject recruitment and sample collection

Our study included 20 healthy samples and 20 COVID-19 samples. The healthy peoples and COVID-19 patients were collected at Nanning First People’s Hospital. A total of 20 COVID-19 patients hospitalized in Nanning First People’s Hospital from December 2022 to January 2023 were selected, and data collection included symptoms, signs, laboratory tests and chest CT examination. The throat swab test was positive for the new coronavirus nucleic acid, and the patients had typical symptoms such as cough and fever, and the clinical classification was mild or medium according to China’s “Diagnosis and Treatment Plan for Novel Coronavirus Infection (Trial Version 10)”. In the control group, 20 healthy people were selected with negative throat swabs for novel coronavirus, no respiratory symptoms, no serious cardiovascular and cerebrovascular diseases, impaired liver and kidney function, diarrhea or constipation. To collect the feces in the middle of the feces, place the container containing the feces in an airtight bag and refrigerate at 4°C. The bag and stool samples are marked with the name and the time the sample was collected. If DNA is extracted on the same day, it can be put at 4°C, extracted within a week, can be put at -20°C, long-term storage needs to be quickly put into liquid nitrogen, and then immediately put into -80°C storage. The study was approved by Medical Ethics Committee of the Fifth Affiliated Hospital of Guangxi Medical University. All patients had signed an informed consent form.

Information on the collection, storage and preparation of samples: collect the feces in the middle of the feces, place the container containing the feces in an airtight bag, and refrigerate at 4°C. The bag and stool samples are marked with the name and the time the sample was collected. If DNA is extracted on the same day, it can be put at 4°C, extracted within a week, can be put at -20°C, long-term storage needs to be quickly put into liquid nitrogen, and then immediately put into -80°C storage.

### 2.2 Microbial 16S rRNA gene sequence analysis

Nucleic acids were extracted using the Surbiopure Fecal Nucleic Acid Extraction Kit (magnetic bead method) (Guangzhou Cybex Biotechnology Co., Ltd.). QubitTM4.0 (Thermo Fisher Scientifi) was used for the extracted DNA, the TransStartFastPfu Fly DNA Polymerase kit (Beijing TransGen Biotech Co., Ltd.) was used by the amplifier MiniAmp Plus Thermal Cycler (Thermo Fisher Scientifi) amplified the V3-V4 region of the bacterial 16S rRNA gene, and the primer group was 341F5’-TCGTCGGCAGGTCAGATGTGTATAAGAGAGAGCCTACGGGGGGWGCAG-3’ and 805R5’-GTCTCGTGGTCGGCGGAGATGTATAAGAGAGGACTACHVGGGTATCTAATCC-3’, Reaction systems and amplification procedures. The product was purified using magnetic DNA Beads (Beijing TransGen Biotech Co., Ltd.). The purified products were quantified using the bioanalyzers Agilent 2100 (Agilent Technologies) and QubitTM4.0 (Thermo Fisher Scientifi). The combined library was sequenced using the Illumina MiSeq instrument, which was packaged using MiSeq Reagent Kit v3 (Illumina, Inc., San Diego, CA, USA).

### 2.3 Bioinformatics analysis

Based on the 16S rRNA sequencing data, the valid sequences of the samples were clustered and grouped into Amplicon Sequence Variant (ASVs) *via* DATA2 in the QIIME2. The ASVs with relative abundance greater than 0.01% in all samples were used for subsequent analysis. Alpha diversity and beta diversity were analyzed to assess whether there was difference in species richness and diversity as well as species composition between COVID-19 and control groups. Alpha diversity was assessed through the observed features index, Evenness index, PD index, and Shannon index. The beta diversity analysis was performed using principal coordinates analysis (PCoA) and non-metric multidimensional scaling (NMDS). The analysis of similarities (ANOSIM) was utilized to compare different groups. At the phylum and genus levels, the abundance of bacterial taxa between COVID-19 and control groups was compared *via* Wilcoxon test. Linear discriminant analysis Effect Size (LEfSe) was utilized to analyze the variability in the abundance of bacterial taxa from different classification levels. The screening criteria were LDA score > 2 and *P <* 0.05. The PICRUSt2 was utilized to perform the enrichment analysis. The differences of enriched pathways between COVID-19 and control groups were compared *via* Wilcoxon test. Afterwards, three machine learning algorithms, namely random forest (RF), least absolute shrinkage and selection operator (LASSO), and univariate logistic regression, were utilized to identify key ASVs. The effectiveness of the three models and diagnostic value of key ASVs for COVID-19 were assessed *via* receiver operating characteristic (ROC) curves. Finally, at the genus level, the correlation of the top30 bacterial taxa was calculated in COVID-19 and control groups using Spearman algorithm, respectively. And the relevant networks were created with |r| > 0.6 and *P <* 0.05 by GgClusterNet (version 0.1.0).

### 2.4 Statistical analysis

Statistical analysis was carried out through R software (version 4.1.1, https://www.r-project.org/). Differences between groups were analyzed *via* the Wilcoxon test. *P <* 0.05 represented a significant difference.

## 3 Results

### 3.1 Taxonomic analysis of intestinal flora in COVID-19 and control groups

**Figure 1:**
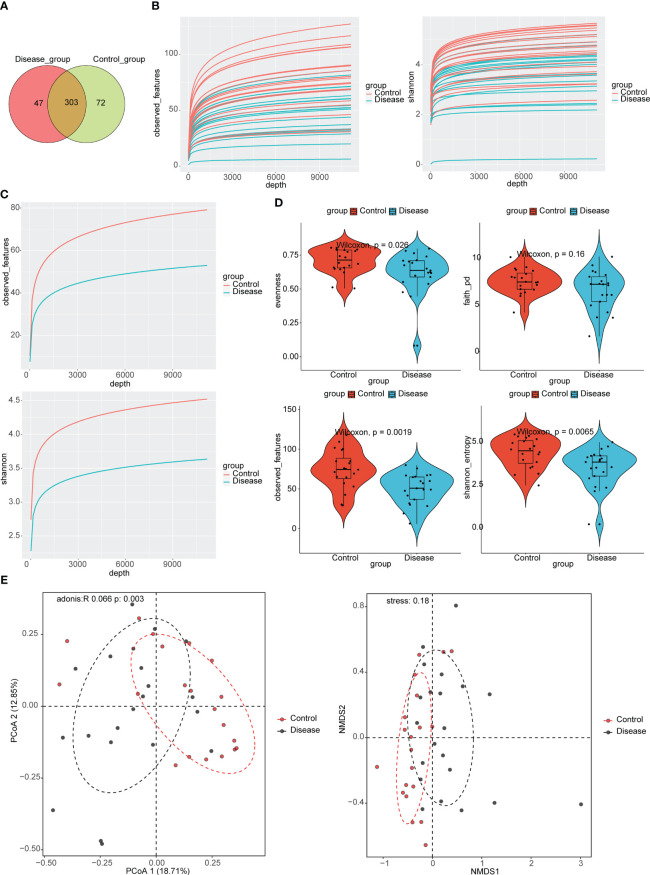
Taxonomic annotation of Intestinal Flora in COVID-19 and control groups. **(A)** Venn diagram for the common Amplicon Sequence Variant (ASVs) in COVID-19 and control groups. **(B)** Analysis of alpha diversity based on the observed features and the Shannon index dilution curves in COVID-19 groups. **(C)** Analysis of alpha diversity based on the observed features and the Shannon index dilution curves in control groups. **(D)** Violin plots for differences of observed features index, Evenness index, PD index, and Shannon index between COVID-19 and control groups. **(E)** Analysis of beta diversity based on principal coordinates analysis (PCoA) and nonmetric multidimensional scaling (NMDS), COVID-19 and control subjects were denoted with black and red nodes, respectively.

In total, 303 ASVs were shared between COVID-19 and control groups, such as ASV1, ASV2, ASV3, etc. (**Supplementary Table 1**; **Figure 1A**). There were 47 ASVs that were specific to the COVID-19 group, such as ASV5, ASV8, ASV9, etc. (**Supplementary Table 1**; **Figure 1A**). There were 72 ASVs that were specific to the control group, such as ASV6, ASV10, ASV29, etc. (**Supplementary Table 1**; **Figure 1A**). The results of alpha diversity analysis showed that the observed features and Shannon dilution curves of the sequencing data had leveled off, indicating that the majority of species had been captured (**Figure 1B**). Furthermore, there were more species in the control group than in COVID-19 group (**Figure 1C**). To further compare the differences in species richness between COVID-19 and control groups, we measured the alpha diversity of species by observed features index, Evenness index, PD index, and Shannon index. The results indicated that the Shannon index, observed features index, and Evenness index in the control groups were markedly higher than in the COVID-19 group (**Figure 1D**). In order to analyze whether there were different in the composition of the bacterial taxa between COVID-19 and control groups, we performed PCoA and NMDS. The results suggested that there were markedly different in bacterial taxa composition between the COVID-19 and control groups (**Figure 1E**).

**Figure 2:**
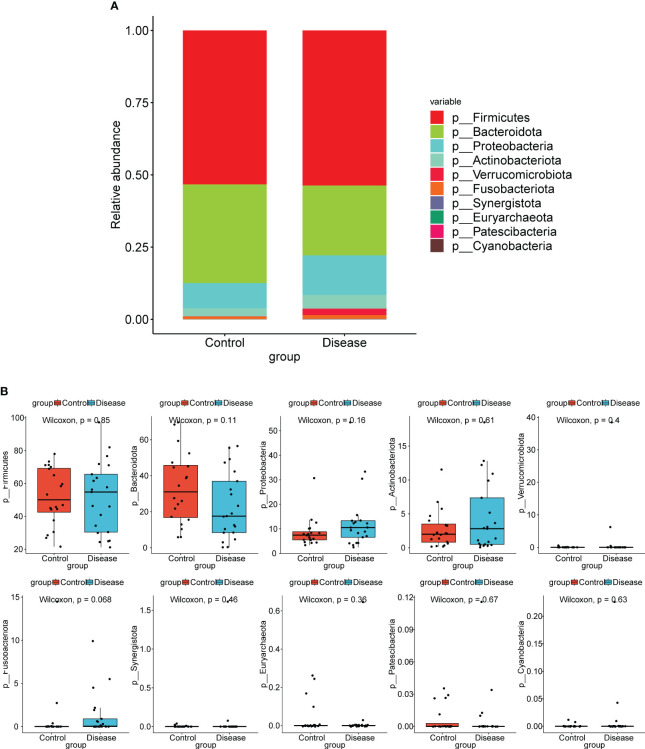
Analysis of species composition diversity at the phylum levels. **(A)** Stacked bar chart for the releative aboundances of the top10 bacterial taxa at the phylum level. **(B)** Box plots for differences of the relative abundance of ten bacterial taxa between COVID-19 and control groups.

At the phylum level, top10 bacterial taxa in terms of relative abundance were shown (**Figure 2A**). The relative abundance of Bacteroidota was higher in control group. The relative abundance of Actinobacteriota, Proteobacteria, and Verrucomicrobiota was higher in the COVID-19 group. However, the differences of bacterial taxa between COVID-19 and control groups were not significant (**Figure 2B**).

**Figure 3:**
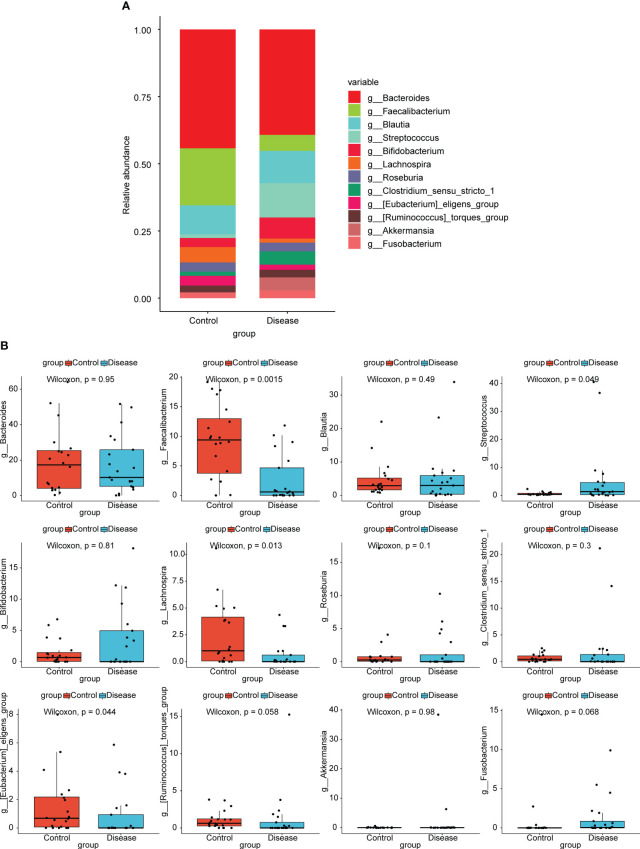
Analysis of species composition diversity at the genus levels. **(A)** Stacked bar chart for the releative aboundances of the top12 bacterial taxa at the genus level. **(B)** Box plots for differences of the relative abundance of 12 bacterial taxa between COVID-19 and control groups.

At the genus level, top12 bacterial taxa in terms of relative abundance were shown (**Figure 3A**). The relative abundance of [*Eubacterium*] *eligens* group, *Bacteroides*, *Lachnospira*, and *Faecalibacterium* was higher in control group. The relative abundance of *Streptococcus*, *Bifidobacterium*, *Clostridium* sensu stricto 1, and *Akkermansia* was higher in COVID-19 group. Additionally, the results of statistical analysis demonstrated that the relative abundance of *Faecalibacterium*, *Streptococcus*, *Lachnospira*, and [*Eubacterium*] *eligens* group between COVID-19 and normal groups was markedly different (**Figure 3B**).

**Figure 4:**
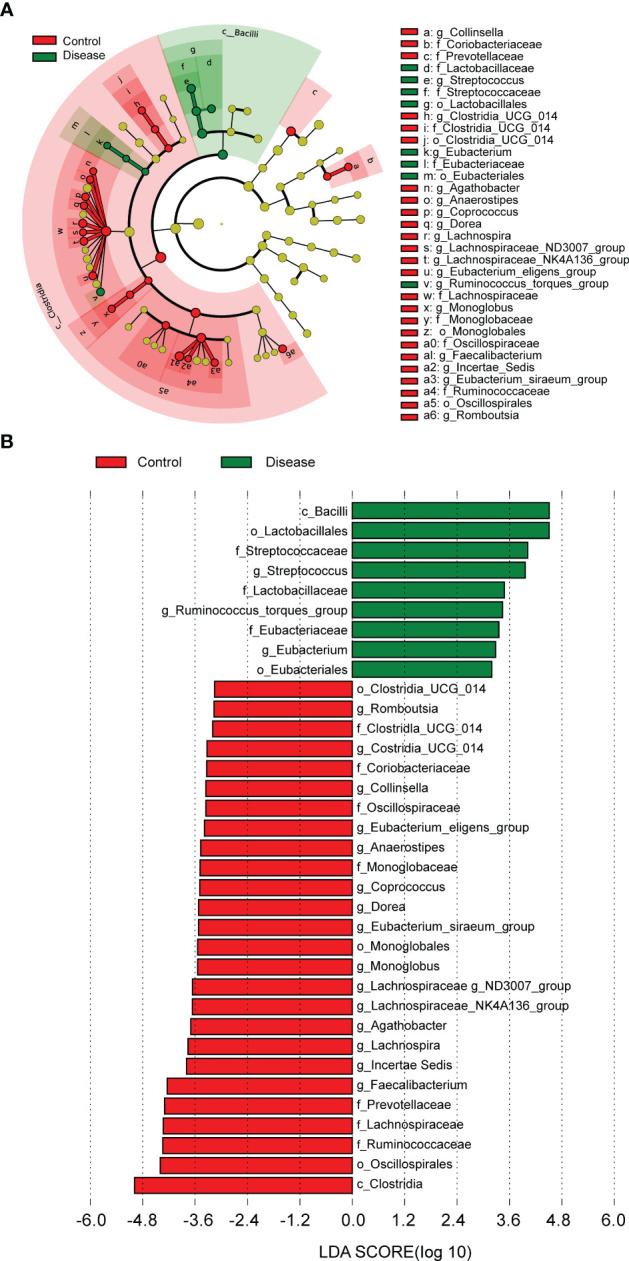
The linear discriminant analysis (LDA) effect size (LEFSe) analysis to screen specific dominant bacterial taxa of the two groups. **(A)** Cladogram of LEFSe analysis demonstrated microbiome differences of the two groups at various phylogenic levels. Circles from the inside out indicate the phylogenetic levels from the phylum to genus. **(B)** Distribution histogram based on LDA analysis (LDA score > 2 and *P <* 0.05). The abscissa represents the LDA score, and the ordinate represents the differential bacterial taxa.

In order to further analyze whether there were differences of specific dominant bacterial taxa between the COVID-19 and control groups, LEfSe was performed. The results showed the nine bacterial taxa were significantly more abundant in the COVID-19 group, such as *g-Streptococcus, f-Streptococcaceae, o-Lactobacillales, c-Bacilli*, and so on (**Figures 4A, B**). In the control group, 26 bacterial taxa were significantly more abundant, such as *c-Clostrjdia, o-Oscillospirales, f-Ruminococcaceae*, etc. (**Figures 4A, B**).

### 3.2 Enrichment analysis

**Figure 5:**
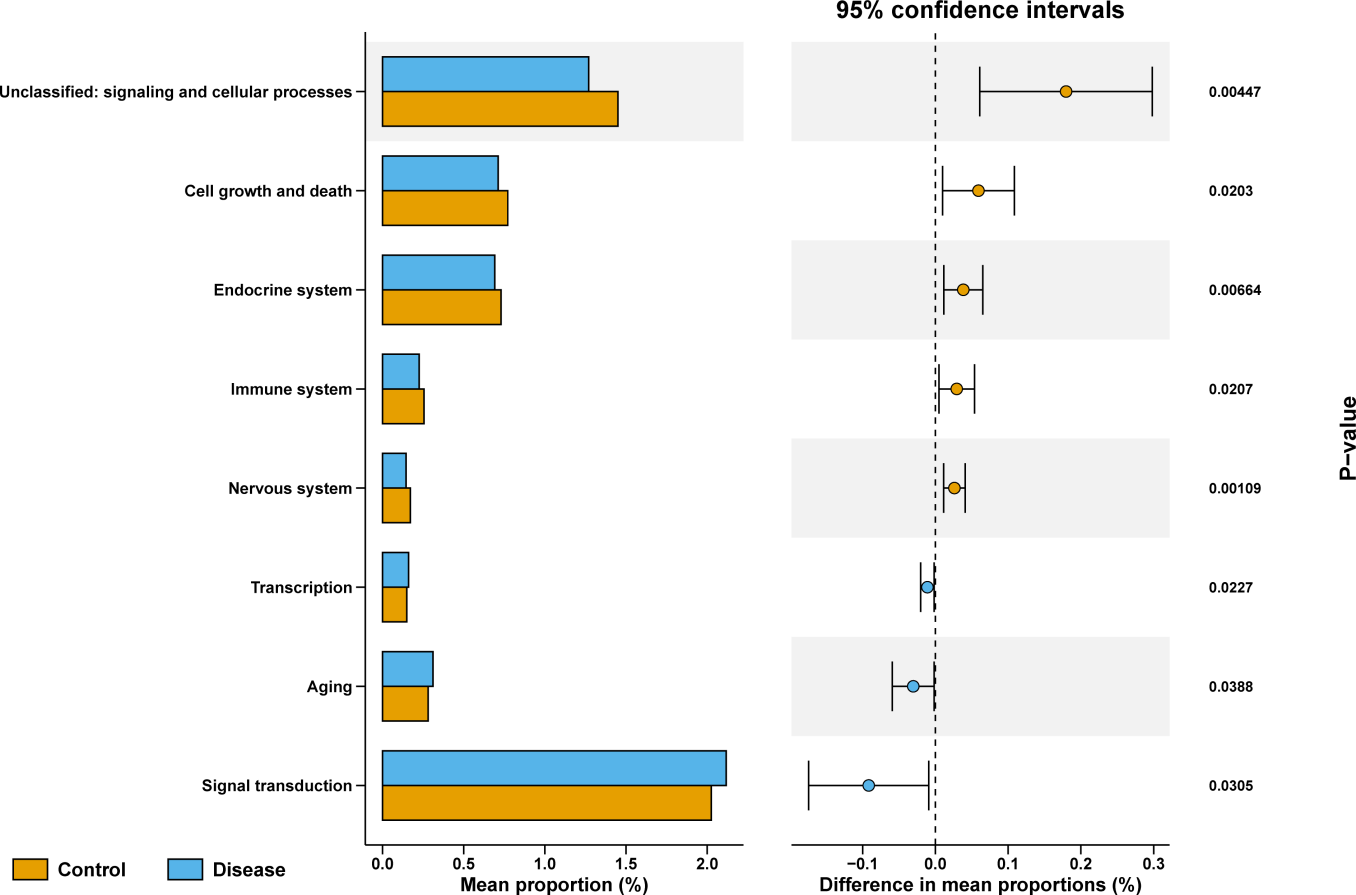
KEGG enriched pathways analysis between COVID-19 and control groups, Data in different groups was analyzed with Wilcoxon test.

To explore the differences in functional characteristics of bacterial taxa between COVID-19 and control groups, we performed KEGG pathways prediction. As shown in **Figure 5**, unclassified: signaling and cellular processes, cell growth and death, endocrine system, immune system, and nervous system were markedly enriched in the control group, while the transcription, aging, and signal transduction were significantly enriched in COVID-19 group.

### 3.3 Acquisition of key ASVs

**Figure 6:**
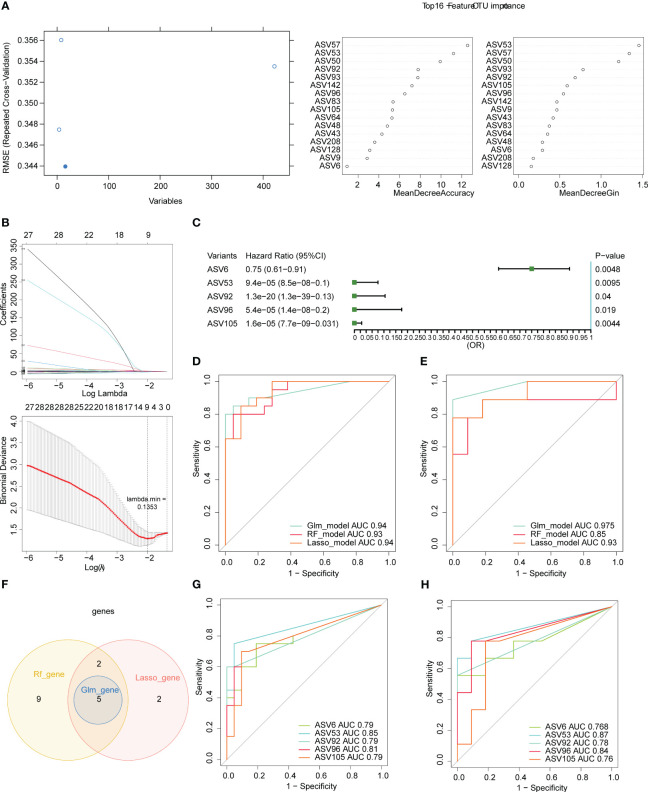
Five key ASVs were screened using three machine learning algorithms. **(A)** Variable importance plot of the random forest (RF) algorithm. **(B)** Least absolute shrinkage and selection operator (LASSO) regression analysis and cross-validation for tuning parameter selection were displayed. **(C)** Forest plot for univariate logistic regression. Receiver operating characteristic (ROC) curves of three models in the training (n= 20, **D**) and validation sets (n= 20, **E**). **(F)** Venn diagram for five key ASVs among in three algorithms. Receiver operating characteristic (ROC) curves of five key ASVs in the training **(G)** and validation **(H)** sets.

To further filter the key ASVs, we randomly selected 20 samples from the 40 samples as the training set and the remaining 20 samples as the validation set. For the RF algorithm, we obtained 16 characteristic ASVs, namely ASV57, ASV53, ASV50, ASV92, ASV93, ASV142, ASV96, ASV83, ASV105, ASV64, ASV48, ASV43, ASV208, ASV128, ASV9, and ASV6 (**Figure 6A**). There were 9 characteristic ASVs *via* LASSO algorithm, containing ASV105, ASV96, ASV181, ASV53, ASV142, ASV92, ASV6, ASV111, and ASV208 (**Figure 6B**). The results of univariate logistic regression demonstrated that the 5 ASVs were protective factors for COVID-19, namely ASV6, ASV53, ASV92, ASV96, and ASV105 (**Figure 6C**). AUC values of three models were greater than 0.85 for both the training and validation sets, suggesting that three models all had good accuracy (**Figures 6D, E**). The 5 key ASVs were obtained through taking the intersection of the characteristic ASVs obtained by the three algorithms, namely ASV6, ASV53, ASV92, ASV96, and ASV105 (**Figure 6F**). Details of the 5 key ASVs were shown in **Table 1**. The AUC values of all key ASVs were greater than 0.7 for both the training and validation sets, indicating that 5 key ASVs had diagnostic value for COVID-19 (**Figures 6G, H**).

**Table 1:** Details for the phylogenetic levels of 5 key ASVs in COVID-19.

OUT	Kingdom	Phylum	Class	Order	Family	Genus	Species
ASV6	d Bacteria	p Firmicutes	c Clostridia	o Oscillospirales	f Ruminococcaceae	g Faecalibacterium	
ASV53	d Bacteria	p Firmicutes	c Clostridia	o Lachnospirales	f Lachnospiraceae	g Coprococcus	
ASV92	d Bacteria	p Firmicutes	c Clostridia	o Oscillospirales	f Oscillospiraceae		
ASV96	d Bacteria	p Firmicutes	c Clostridia	o Lachnospirales	f Lachnospiraceae	g Lachnospiraceae_ND3007_group	s metagenome
ASV105	d Bacteria	p Firmicutes	c Clostridia	o Lachnospirales	f Lachnospiraceae		

### 3.4 Creation of relevance networks

**Figure 7:**
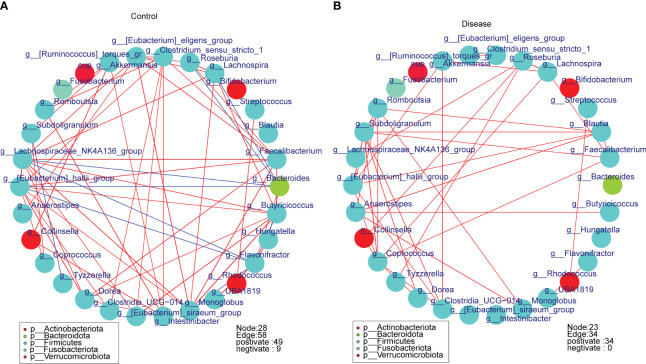
The spearman correlation networks of the top30 bacterial taxa in different groups at the genus level. **(A)** Control group, **(B)** COVID-19 group.

To further assess the correlation between bacterial taxa, we constructed relevance networks in COVID-19 and control groups, separately. The relevance network in the control group contained 28 nodes and 58 edges (**Figure 7A**). The *g*-*Lachnospiraceae* NK4A136 group associated with multiple bacterial taxa. For instance, it was negatively correlated with *g*-*Bacteroides* and *g*-*Flavonifractor*, but positively correlated with *g-Faecalibacterium* and *g-Lachnospira*, etc. In the COVID-19 group, the relevance network contained 23 nodes and 34 edges (**Figure 7B**). The *g-Romboutsia* was positively relevant to multiple bacterial taxa, such as *g-Blautia, g-Monoblobus, g-Coprococeus*, and so on. In conclusion, the relevance network in the control group was more complex compared to the COVID-19 group.

## 4 Discussion

Since December 2019, coronavirus disease 2019 (COVID-19), caused by severe acute respiratory syndrome coronavirus type 2 (SARS-CoV-2), has spread rapidly globally and evolved into the worst pandemic in the last 100 years. In previous studies, it has been confirmed that the intestinal flora has an influence and regulating effect on respiratory disease (Ichinohe et al., 2011; Fagundes et al., 2012). In animal models, the gut microbiota affects the prognosis of infectious lung disease (Schuijt et al., 2016), and similarly, lung infections affect the gut microbiota (Wang et al., 2014), suggesting a two-way crosstalk between the gut and lungs (Sencio et al., 2021).

Previous studies have found that respiratory viral infections affect systemic microbiota dynamics and lead to quantitative intestinal dysbiosis (Yildiz et al., 2018). For example,

In a study for the K18-hACE2 transgenic mouse model infected with SARS-CoV-2, the cecal microbiome showed a decrease in the Shannon index, and the degree of reduction correlated with the dose of SARS-CoV-2 infection (Seibert et al., 2021). Tatiana et al. compared the gut microbiota profile of COVID-19 individuals at day 0 (inclusion) and day 7 using 16S metagenomics data, and revealed that although similar at inclusion, Shannon alpha diversity index significantly decreased in COVID-19 and non-COVID-19 groups than in the control group at day 7 (Galperine et al., 2023). Another study conducted using shotgun sequencing showed that, though the overall gut microbiota composition differed between COVID-19 patients and non-subjects, there were no significant differences in species richness and Shannon diversity (Yeoh et al., 2021). Besides, 16S rRNA sequencing data of the intestinal microbiome of patients with active SARS-CoV-2 infection were analyzed and compared with those of recovered patients and uninfected healthy controls. The results showed that except for the Pielou evenness of COVID-19 positive patients, the use of antibiotics was associated with a moderate decrease in OTUs and Shannon index (p = 0.05) (Yin et al., 2022). It was reported that vaccination could significantly inhibit the shannon, pielou evenness, simpson and invsimpson index, as characterized by COVID-19 infection, which may benefit the host immune response to prevent COVID-19 (Jiao et al., 2022).

In our study, the control group exhibited higher microbiota diversity and richness than the COVID-19 group, there were higher values on the α diversity analysis, the observation characteristic index, Shannon index and uniformity index in the control group compared to the COVID-19 group. This is consistent with previous research. It is worth that, although one study indicated that ethnic, regional factors, and socioeconomic characteristics are considered as independent major factors influencing the gut microbiome(Brooks et al., 2018), and the regional factors have the greatest influence on gut microbiome composition (Ghosh et al., 2020), several studies from both Europe and Asia suggest that the diversity of gut microbial in COVID-19 patients is significantly reduced, and there are significant differences in gut microbiome composition compared to controls. For example, in a cohort of European populations consisting mainly of patients of Caucasian ethnicity, it was found that the gut microbiota of patients infected with SARS-CoV-2 was significantly different from that of patients with SARS-CoV-2 negative (Reinold et al., 2021). A study from Portugal has proposed for the first time that gut microbiota diversity is a risk prognostic biomarker for COVID-19 severity with moderate and severe COVID-19 inpatients having a lower Shannon diversity index (Moreira-Rosário et al., 2021). Besides, a Chinese study also found that patients suffering COVID-19 infection had significantly lower gut microbiota diversity compared to healthy controls or seasonal influenza patients, and changes in gut microbiota composition could lead to cytokine storms (Tao et al., 2020).

Next, we performed PCoA and NMDS and observed that there were significant differences in bacterial taxa composition between the COVID-19 and control groups. Specifically speaking, at the phylum levels, the controls had a higher relative abundance of *Bacteroides*, and the relative abundance of *actinobacillus*, *proteus*, and verrucous microbiota was higher in the COVID-19 group. At the genus level, the relative abundance of [*EuBacteroides*] eligens group, *Bacteroides*, *Lactobacillus*, and *Faecal bacillus* was higher in the control group. Relative abundance was higher in the COVID-19 group of *streptococci*, *bifidobacteria*, *Clostridium strict-sensitizer1*, and *Akmancia*. Several literature have extended the theoretical basis for these results. In Zuo’s study, the abundance of *fecal bacilli* is inversely correlated with disease severity (Zuo et al., 2020), consistent with the lower expression of fecal bacteria in the COVID-19 group compared to controls. And meanwhile, according to the findings of Yeoh and Reinold, at the phylum level, members of *Bacteroides* were relatively abundant in COVID-19 patients compared to non-COVID-19 individuals, while Actinomycetes were relatively abundant in non-COVID-19 individuals. In the absence of controlled antibiotic use, differences in the composition of the COVID-19 gut microbiota are mainly enriched by species including *Bifidobacterium juveniles* and *Faecalella przewalski* (Reinold et al., 2021; Yeoh et al., 2021). Considering that not enough non-COVID-19 samples were collected for analysis in this study, these studies provide us with more perspectives targeting the comparing of bacterial taxa composition among controls, COVID-19, and non-COVID-19 individuals. In general, *Bacteroides* was the most abundant in the control group, followed by COVID patients, and finally non-COVID patients. The abundance of Actinomycetes in COVID and non-COVID patients was higher than that in the control group, and the abundance of non-COVID patients increased the most.

In order to analyze the difference in functional characteristics between different groups, we used PICRUSt2 to predict the function of the microbial community. The results of the functional difference analysis of the two groups showed that a total of 8 KEGG pathways were significantly different between the disease and the control group, among which transcription, aging, and signal transduction entries were highly expressed in the disease group, and not classified: signal and cell processes, cell growth and death, endocrine system, immune system, and nervous system were highly expressed in the control group. There are currently few studies on the functional characteristics of COVID-19, and our study is consistent with the Koo H study, which does not share a KEGG metabolic pathway between COVID-19 patients and controls (Koo and Morrow, 2022). While, considering the mechanisms responsible for the high expression of these pathways in COVID-19 patients are unclear, further study was required. Besides, it is as expected that the microbial network in the control group was more complex than in the disease group, and the number of edges and nodes of the microbial network in the control group was greater than that in the disease group.

On the other hands, LASSO, RF, and GLM algorithms were used to screen the five key ASVs. as the best model: ASV6, ASV53, ASV92, ASV96, ASV105 belonged to the Firmicutes phylum, belonging to the genera g*-Faecalibacterium*, g*-Coprococcus* and g*-Lachnospiraceae*_ND3007_group. The Firmicutes/Bacteroides (F/B) ratio is an important indicator of structural changes in the gut microbiota (Mir et al., 2019). Some studies have found high levels of certain gut flora belong to the firmicutes in COVID-19 patients. For example, in a study characterizing the entero-mammary microbiota of women with the presence of the virus during childbirth, several bacterial taxa were high in maternal rectal swab (MRS) positive for SARS-CoV-2 RNA. Most of these bacteria belong to the phylum Firmicutes, such as *bifidobacteriaceae, oscoprospirae*, and *microbacilliae* (Juárez-Castelán et al., 2022). Besides, in the gut of patients with acute post-COVID-19 syndrome, high levels of *Rumen gunavis* have been detected (Liu et al., 2022). Other studies that do not agree with our findings have shown that stool samples with low or zero infectious characteristics of SARS-CoV-1 have a high level of *Spirochetidae bacteria 1_57 *(Zuo et al., 2021). In another study, in COVID-19-positive patients, consumption of the Rumen family was observed (Yin et al., 2022). These studies have shown the operability of the above diagnostic markers for the detection of intestinal microbiome changes and even COVID-19 infection.

This study comprehensively investigated the relationship between the intestinal microbiota and COVID-19 infection using the 16S rRNA sequencing. The relationship between the abundance and diversity of gut flora and COVID-19 were confirmed, and the dominant flora in the intestines of COVID-19 patients were analyzed, as well as the function prediction of these intestinal flora in COVID-19 was also carried out. Further, five diagnostic ASVs were screened for predicting COVID-19 well. All of this provides new ideas for further understanding of the mechanisms by which COVID-19 occurs.

However, our research needs to go further to investigate the mechanisms affecting the function of the gut microbiota, and to analyze it in combination with other groups to further explore the mechanism of COVID-19 development and development. At present, the literature information related to COVID-19 of various flora and the literature of various functional enrichment pathways related to COVID-19 are relatively lacking. It is necessary to verify the exact mechanisms of the enrichment of dominant bacterial taxa in COVID-19 patients through more in-depth *in vivo* and *in vivo* functional experiments. As a single-center study with a limited sample size, the gut microbiota of patients is affected by many factors, such as a decrease in the gut microbiome of the elderly population (Kim et al., 2019).Coinfection and superinfection are common in respiratory viral infections (Zimmermann and Curtis, 2020). When conducting research, it is not possible to completely avoid the different microbiota caused by factors such as different ages, underlying diseases, co-infections, and severity of the disease. Further multicentre studies with the larger size and longer duration and more key factors are warranted in future studies. Last but not least, considering the use of healthy samples can be more focused on the impact of COVID-19 infection on normal biological status and investigate the biology and mechanisms of COVID-19 infection, and due to data availability limitations, we were temporarily unable to obtain a sufficient number of samples from non-COVID-19 infection for analysis. We will try to obtain more samples of non-COVID-19 infection depending on the cooperation of other research institutions, hospitals or clinical trials for a more comprehensive comparative analysis in follow-up studies.

## Data availability statement

The datasets presented in this study can be found in online repositories. The names of the repository/repositories and accession number(s) can be found below: NCBI, PRJNA996048.

## Ethics statement

The studies involving humans were approved by Medical Ethics Committee of the Fifth Affiliated Hospital of Guangxi Medical University. The studies were conducted in accordance with the local legislation and institutional requirements. The participants provided their written informed consent to participate in this study. Written informed consent was obtained from the individuals for the publication of any potentially identifiable images or data included in this article.

## Author contributions

GH: Conceptualization, Data curation, Formal Analysis, Supervision, Writing – original draft. YM: Writing – original draft, Data curation. WZ: Methodology, Writing – original draft. QL: Writing – review & editing, Formal Analysis. RX: Formal Analysis, Writing – review & editing. DH: Conceptualization, Writing – review & editing. YL: Writing – review & editing.

## References

[B1] Aishwarya S. Gunasekaran K. (2022). Meta-analysis of the microbial biomarkers in the gut-lung crosstalk in COVID-19, community-acquired pneumonia and Clostridium difficile infections. Lett. Appl. Microbiol. 75 (5), 1293–1306. doi: 10.1111/lam.13798 35920823 PMC9539240

[B2] Alharbi K. S. Singh Y. Hassan Almalki W. Rawat S. Afzal O. Alfawaz Altamimi A. S.. (2022). Gut Microbiota Disruption in COVID-19 or Post-COVID Illness Association with severity biomarkers: A Possible Role of Pre/Pro-biotics in manipulating microflora. Chem. Biol. Interact. 358, 109898. doi: 10.1016/j.cbi.2022.109898 35331679 PMC8934739

[B3] Brooks A. W. Priya S. Blekhman R. Bordenstein S. R. (2018). Gut microbiota diversity across ethnicities in the United States. PloS Biol. 16 (12), e2006842. doi: 10.1371/journal.pbio.2006842 30513082 PMC6279019

[B4] Cao J. Wang C. Zhang Y. Lei G. Xu K. Zhao N.. (2021). Integrated gut virome and bacteriome dynamics in COVID-19 patients. Gut Microbes 13 (1), 1–21. doi: 10.1080/19490976.2021.1887722 PMC7946006 33678150

[B5] Du M. Cai G. Chen F. Christiani D. C. Zhang Z. Wang M. (2020). Multiomics evaluation of gastrointestinal and other clinical characteristics of COVID-19. Gastroenterology 158 (8), 2298–2301.e2297. doi: 10.1053/j.gastro.2020.03.045 32234303 PMC7270476

[B6] Fagundes C. T. Amaral F. A. Vieira A. T. Soares A. C. Pinho V. Nicoli J. R.. (2012). Transient TLR activation restores inflammatory response and ability to control pulmonary bacterial infection in germfree mice. J. Immunol. 188 (3), 1411–1420. doi: 10.4049/jimmunol.1101682 22210917

[B7] Gaibani P. D’Amico F. Bartoletti M. Lombardo D. Rampelli S. Fornaro G.. (2021). The gut microbiota of critically ill patients with COVID-19. Front. Cell Infect. Microbiol. 11. doi: 10.3389/fcimb.2021.670424 PMC8276076 34268136

[B8] Galperine T. Choi Y. Pagani J. L. Kritikos A. Papadimitriou-Olivgeris M. Méan M.. (2023). Temporal changes in fecal microbiota of patients infected with COVID-19: a longitudinal cohort. BMC Infect. Dis. 23 (1), 537. doi: 10.1186/s12879-023-08511-6 37596518 PMC10436399

[B9] Ghosh T. S. Das M. Jeffery I. B. O’Toole P. W. (2020). Adjusting for age improves identification of gut microbiome alterations in multiple diseases. Elife 9, e50240. doi: 10.7554/eLife.50240 32159510 PMC7065848

[B10] Golonka R. Yeoh B. S. Vijay-Kumar M. (2019). Dietary additives and supplements revisited: the fewer, the safer for liver and gut health. Curr. Pharmacol. Rep. 5 (4), 303–316. doi: 10.1007/s40495-019-00187-4 32864300 PMC7453625

[B11] Gu J. Han B. Wang J. (2020). COVID-19: gastrointestinal manifestations and potential fecal-oral transmission. Gastroenterology 158 (6), 1518–1519. doi: 10.1053/j.gastro.2020.02.054 32142785 PMC7130192

[B12] Guan W. J. Ni Z. Y. Hu Y. Liang W. H. Ou C. Q. He J. X.. (2020). Clinical characteristics of coronavirus disease 2019 in China. N. Engl. J. Med. 382 (18), 1708–1720. doi: 10.1056/NEJMoa2002032 32109013 PMC7092819

[B13] Ichinohe T. Pang I. K. Kumamoto Y. Peaper D. R. Ho J. H. Murray T. S.. (2011). Microbiota regulates immune defense against respiratory tract influenza A virus infection. Proc. Natl. Acad. Sci. U.S.A. 108 (13), 5354–5359. doi: 10.1073/pnas.1019378108 21402903 PMC3069176

[B14] Jiao J. Shen Y. Wang P. Zuo K. Yang X. Chen M.. (2022). Characterization of the intestinal microbiome in healthy adults over Sars-Cov-2 vaccination. Front. Biosci. (Landmark Ed) 27 (10), 280. doi: 10.31083/j.fbl2710280 36336856

[B15] Johnson C. H. Spilker M. E. Goetz L. Peterson S. N. Siuzdak G. (2016). Metabolite and microbiome interplay in cancer immunotherapy. Cancer Res. 76 (21), 6146–6152. doi: 10.1158/0008-5472.Can-16-0309 27729325 PMC5093024

[B16] Juárez-Castelán C. J. Vélez-Ixta J. M. Corona-Cervantes K. Piña-Escobedo A. Cruz-Narváez Y. Hinojosa-Velasco A.. (2022). The entero-mammary pathway and perinatal transmission of gut microbiota and SARS-CoV-2. Int. J. Mol. Sci. 23 (18), 10306. doi: 10.3390/ijms231810306 36142219 PMC9499685

[B17] Keely S. Talley N. J. Hansbro P. M. (2012). Pulmonary-intestinal cross-talk in mucosal inflammatory disease. Mucosal Immunol. 5 (1), 7–18. doi: 10.1038/mi.2011.55 22089028 PMC3243663

[B18] Kim B. S. Choi C. W. Shin H. Jin S. P. Bae J. S. Han M.. (2019). Comparison of the gut microbiota of centenarians in longevity villages of South Korea with those of other age groups. J. Microbiol. Biotechnol. 29 (3), 429–440. doi: 10.4014/jmb.1811.11023 30661321

[B19] Koo H. Morrow C. D. (2022). Early indicators of microbial strain dysbiosis in the human gastrointestinal microbial community of certain healthy humans and hospitalized COVID-19 patients. Sci. Rep. 12 (1), 6562. doi: 10.1038/s41598-022-10472-w 35449389 PMC9022020

[B20] Liu Q. Mak J. W. Y. Su Q. Yeoh Y. K. Lui G. C. Ng S. S. S.. (2022). Gut microbiota dynamics in a prospective cohort of patients with post-acute COVID-19 syndrome. Gut 71 (3), 544–552. doi: 10.1136/gutjnl-2021-325989 35082169

[B21] Merenstein C. Liang G. Whiteside S. A. Cobián-Güemes A. G. Merlino M. S. Taylor L. J.. (2022). Correction for Merenstein et al., “Signatures of COVID-19 Severity and Immune Response in the Respiratory Tract Microbiome”. mBio 13 (5), e0229322. doi: 10.1128/mbio.02293-22 36043873 PMC9601094

[B22] Mir R. A. Kleinhenz M. D. Coetzee J. F. Allen H. K. Kudva I. T. (2019). Fecal microbiota changes associated with dehorning and castration stress primarily affects light-weight dairy calves. PloS One 14 (1), e0210203. doi: 10.1371/journal.pone.0210203 30673718 PMC6344101

[B23] Moreira-Rosário A. Marques C. Pinheiro H. Araújo J. R. Ribeiro P. Rocha R.. (2021). Gut microbiota diversity and C-Reactive protein are predictors of disease severity in COVID-19 patients. Front. Microbiol. 12. doi: 10.3389/fmicb.2021.705020 PMC8326578 34349747

[B24] Onder G. Rezza G. Brusaferro S. (2020). Case-Fatality rate and characteristics of patients dying in relation to COVID-19 in Italy. Jama 323 (18), 1775–1776. doi: 10.1001/jama.2020.4683 32203977

[B25] Park D. H. Kothari D. Niu K. M. Han S. G. Yoon J. E. Lee H. G.. (2019). Effect of fermented medicinal plants as dietary additives on food preference and fecal microbial quality in dogs. Anim. (Basel) 9 (9), 690. doi: 10.3390/ani9090690 PMC6770862 31527540

[B26] Peters K. M. Carlson B. A. Gladyshev V. N. Tsuji P. A. (2018). Selenoproteins in colon cancer. Free Radic. Biol. Med. 127, 14–25. doi: 10.1016/j.freeradbiomed.2018.05.075 29793041 PMC6168369

[B27] Reinold J. Farahpour F. Fehring C. Dolff S. Konik M. Korth J.. (2021). A pro-inflammatory gut microbiome characterizes SARS-CoV-2 infected patients and a reduction in the connectivity of an anti-inflammatory bacterial network associates with severe COVID-19. Front. Cell Infect. Microbiol. 11. doi: 10.3389/fcimb.2021.747816 PMC8635721 34869058

[B28] Ren Z. Wang H. Cui G. Lu H. Wang L. Luo H.. (2021). Alterations in the human oral and gut microbiomes and lipidomics in COVID-19. Gut 70 (7), 1253–1265. doi: 10.1136/gutjnl-2020-323826 33789966 PMC8042598

[B29] Schuijt T. J. Lankelma J. M. Scicluna B. P. de Sousa e Melo F. Roelofs J. J. de Boer J. D.. (2016). The gut microbiota plays a protective role in the host defence against pneumococcal pneumonia. Gut 65 (4), 575–583. doi: 10.1136/gutjnl-2015-309728 26511795 PMC4819612

[B30] Seibert B. Cáceres C. J. Cardenas-Garcia S. Carnaccini S. Geiger G. Rajao D. S.. (2021). Mild and severe SARS-CoV-2 infection induces respiratory and intestinal microbiome changes in the K18-hACE2 transgenic mouse model. Microbiol. Spectr. 9 (1), e0053621. doi: 10.1128/Spectrum.00536-21 34378965 PMC8455067

[B31] Sencio V. MaChado M. G. Trottein F. (2021). The lung-gut axis during viral respiratory infections: the impact of gut dysbiosis on secondary disease outcomes. Mucosal Immunol. 14 (2), 296–304. doi: 10.1038/s41385-020-00361-8 33500564 PMC7835650

[B32] Tao W. Zhang G. Wang X. Guo M. Zeng W. Xu Z.. (2020). Analysis of the intestinal microbiota in COVID-19 patients and its correlation with the inflammatory factor IL-18. Med. Microecol. 5, 100023. doi: 10.1016/j.medmic.2020.100023 34173452 PMC7832617

[B33] Wang J. Li F. Wei H. Lian Z. X. Sun R. Tian Z. (2014). Respiratory influenza virus infection induces intestinal immune injury *via* microbiota-mediated Th17 cell-dependent inflammation. J. Exp. Med. 211 (12), 2397–2410. doi: 10.1084/jem.20140625 25366965 PMC4235643

[B34] Xiao F. Tang M. Zheng X. Liu Y. Li X. Shan H. (2020). Evidence for gastrointestinal infection of SARS-CoV-2. Gastroenterology 158 (6), 1831–1833.e1833. doi: 10.1053/j.gastro.2020.02.055 32142773 PMC7130181

[B35] Yang Z. Liu Y. Wang L. Lin S. Dai X. Yan H.. (2022). Traditional Chinese medicine against COVID-19: Role of the gut microbiota. BioMed. Pharmacother. 149, 112787. doi: 10.1016/j.biopha.2022.112787 35279010 PMC8901378

[B36] Yeoh Y. K. Zuo T. Lui G. C. Zhang F. Liu Q. Li A. Y.. (2021). Gut microbiota composition reflects disease severity and dysfunctional immune responses in patients with COVID-19. Gut 70 (4), 698–706. doi: 10.1136/gutjnl-2020-323020 33431578 PMC7804842

[B37] Yildiz S. Mazel-Sanchez B. Kandasamy M. Manicassamy B. Schmolke M. (2018). Influenza A virus infection impacts systemic microbiota dynamics and causes quantitative enteric dysbiosis. Microbiome 6 (1), 9. doi: 10.1186/s40168-017-0386-z 29321057 PMC5763955

[B38] Yin Y. S. Minacapelli C. D. Parmar V. Catalano C. C. Bhurwal A. Gupta K.. (2022). Alterations of the fecal microbiota in relation to acute COVID-19 infection and recovery. Mol. BioMed. 3 (1), 36. doi: 10.1186/s43556-022-00103-1 36437420 PMC9702442

[B39] Zimmermann P. Curtis N. (2020). Coronavirus infections in children including COVID-19: an overview of the epidemiology, clinical features, diagnosis, treatment and prevention options in children. Pediatr. Infect. Dis. J. 39 (5), 355–368. doi: 10.1097/inf.0000000000002660 32310621 PMC7158880

[B40] Zuo T. Liu Q. Zhang F. Lui G. C. Tso E. Y. Yeoh Y. K.. (2021). Depicting SARS-CoV-2 faecal viral activity in association with gut microbiota composition in patients with COVID-19. Gut 70 (2), 276–284. doi: 10.1136/gutjnl-2020-322294 32690600 PMC7385744

[B41] Zuo T. Zhang F. Lui G. C. Y. Yeoh Y. K. Li A. Y. L. Zhan H.. (2020). Alterations in gut microbiota of patients with COVID-19 during time of hospitalization. Gastroenterology 159 (3), 944–955.e948. doi: 10.1053/j.gastro.2020.05.048 32442562 PMC7237927

