# Supplementary material for: Explore the changes of intestinal flora in patients with coronavirus disease 2019 based on bioinformatics
Source: Front Cell Infect Microbiol. 2023 Oct 13;13:1265028. doi: 10.3389/fcimb.2023.1265028 (PMC10611479; doi:10.3389/fcimb.2023.1265028)
Supplement: Supplementary file 1 [file Table_1.docx]

Explore the changes of intestinal flora in patients with coronavirus disease 2019 (COVID-19) based on bioinformatics

# Supplementary Table

**Supplementary Table 1.** Lists of amplicon sequence variant (ASVs) shared by COVID-19 and control groups and specific to the COVID-19 and control groups.

| **Croups** | **Amplicon Sequence Variant (ASVs)** |
| --- | --- |
| Disease_group﹎Control_group | ASV1, ASV2, ASV3, ASV4, ASV7, ASV11, ASV12, ASV13, ASV14, ASV15, ASV17, ASV18, ASV20, ASV22, ASV24, ASV25, ASV26, ASV27, ASV28, ASV30, ASV32, ASV34, ASV38, ASV39, ASV40, ASV41, ASV44, ASV46, ASV55, ASV56, ASV58, ASV59, ASV60, ASV62, ASV64, ASV65, ASV66, ASV67, ASV69, ASV71, ASV72, ASV73, ASV75, ASV77, ASV78, ASV79, ASV81, ASV82, ASV84, ASV85, ASV86, ASV94, ASV95, ASV98, ASV100, ASV104, ASV106, ASV110, ASV112, ASV117, ASV119, ASV127, ASV133, ASV167, ASV168, ASV172, ASV175, ASV176, ASV178, ASV179, ASV180, ASV182, ASV183, ASV184, ASV185, ASV186, ASV187, ASV188, ASV189, ASV190, ASV191, ASV192, ASV193, ASV194, ASV195, ASV196, ASV197, ASV198, ASV199, ASV200, ASV201, ASV202, ASV203, ASV204, ASV205, ASV206, ASV207, ASV208, ASV209, ASV210, ASV211, ASV212, ASV213, ASV214, ASV216, ASV217, ASV218, ASV219, ASV220, ASV221, ASV222, ASV223, ASV224, ASV225, ASV227, ASV228, ASV229, ASV230, ASV231, ASV232, ASV233, ASV234, ASV235, ASV236, ASV237, ASV238, ASV239, ASV240, ASV241, ASV242, ASV243, ASV244, ASV245, ASV246, ASV247, ASV248, ASV249, ASV250, ASV251, ASV252, ASV253, ASV254, ASV257, ASV258, ASV259, ASV260, ASV262, ASV263, ASV264, ASV265, ASV266, ASV267, ASV268, ASV269, ASV270, ASV271, ASV272, ASV273, ASV274, ASV275, ASV276, ASV277, ASV278, ASV279, ASV280, ASV281, ASV282, ASV283, ASV284, ASV285, ASV286, ASV287, ASV288, ASV289, ASV290, ASV291, ASV292, ASV293, ASV294, ASV295, ASV296, ASV297, ASV298, ASV299, ASV300, ASV301, ASV303, ASV304, ASV305, ASV309, ASV310, ASV311, ASV312, ASV313, ASV314, ASV315, ASV316, ASV317, ASV318, ASV319, ASV320, ASV321, ASV322, ASV323, ASV324, ASV325, ASV326, ASV327, ASV328, ASV329, ASV330, ASV331, ASV332, ASV333, ASV334, ASV335, ASV336, ASV337, ASV338, ASV339, ASV340, ASV341, ASV342, ASV343, ASV344, ASV345, ASV346, ASV347, ASV348, ASV349, ASV350, ASV351, ASV352, ASV353, ASV354, ASV355, ASV356, ASV357, ASV358, ASV359, ASV360, ASV361, ASV362, ASV363, ASV364, ASV365, ASV366, ASV367, ASV368, ASV369, ASV370, ASV371, ASV372, ASV373, ASV374, ASV375, ASV376, ASV377, ASV378, ASV379, ASV380, ASV381, ASV382, ASV383, ASV384, ASV385, ASV386, ASV387, ASV388, ASV389, ASV390, ASV391, ASV392, ASV393, ASV394, ASV395, ASV396, ASV397, ASV398, ASV399, ASV400, ASV401, ASV402, ASV403, ASV404, ASV405, ASV406, ASV407, ASV408, ASV409, ASV410, ASV411, ASV412, ASV413, ASV414, ASV416, ASV417, ASV418, ASV419, ASV421, ASV422, ASV423, ASV430 |
| Control_group | ASV6, ASV10, ASV29, ASV33, ASV36, ASV37, ASV42, ASV43, ASV45, ASV47, ASV48, ASV49, ASV50, ASV53, ASV57, ASV61, ASV68, ASV76, ASV80, ASV83, ASV87, ASV89, ASV91, ASV92, ASV93, ASV96, ASV97, ASV101, ASV102, ASV103, ASV105, ASV108, ASV109, ASV111, ASV113, ASV118, ASV120, ASV121, ASV123, ASV124, ASV125, ASV128, ASV129, ASV131, ASV134, ASV135, ASV136, ASV139, ASV140, ASV141, ASV142, ASV144, ASV147, ASV148, ASV149, ASV151, ASV153, ASV155, ASV157, ASV158, ASV159, ASV160, ASV162, ASV164, ASV166, ASV173, ASV174, ASV181, ASV261, ASV306, ASV307, ASV308 |
| Disease_group | ASV5, ASV8, ASV9, ASV16, ASV19, ASV21, ASV23, ASV31, ASV35, ASV51, ASV52, ASV54, ASV63, ASV70, ASV74, ASV88, ASV90, ASV99, ASV107, ASV114, ASV115, ASV116, ASV122, ASV126, ASV130, ASV132, ASV137, ASV138, ASV143, ASV145, ASV146, ASV150, ASV152, ASV154, ASV156, ASV161, ASV163, ASV165, ASV169, ASV170, ASV171, ASV177, ASV215, ASV226, ASV255, ASV256, ASV302 |
